# Isolation of antimicrobial resistant bacteria in upper respiratory tract infections of patients

**DOI:** 10.1007/s13205-016-0473-z

**Published:** 2016-08-11

**Authors:** Li-min Wang, Xiao-liang Qiao, Liang Ai, Jing-jing Zhai, Xue-xia Wang

**Affiliations:** Department of Clinical Laboratory, Women and Infants Hospital of Zhengzhou, No. 41, Jinshui Road, Jinshui District, Zhengzhou, Henan 450012 People’s Republic of China

**Keywords:** Isolation, Antibiotic resistance, Upper respiratory tract infection, Biofilm

## Abstract

*Haemophilus influenzae*, *Streptococcus pyogenes*, *Moraxella catarrhalis*, *Staphylococcus aureus*, and *Streptococcus pneumoniae* are usual cause of upper respiratory tract infection cases. The present study aims the isolation of bacterial strains which are resistant to the commonly prescribed antibiotics. In total, 900 throat swabs were obtained from the patients suffering from upper respiratory tract infections residing in three different localities. The maximum number of isolates (64 %) were obtained from locality-1 (L-1), whereas lowest isolates were found in second locality (L-2). *H. influenzae* was found to be the most dominant bacterial pathogen in upper respiratory tract infections in patients with 42 % of the total isolates. *H. influenzae* and *Chlamydia pneumoniae* were resistant to β-lactam antibiotics but susceptible to fluroquinolones and aminoglycosides, whereas *S. aureus* and *S. pneumoniae* were found to be highly resistant to β-lactam, aminoglycosides and fluroquinolones. *S. aureus* was also moderately resistant to fluroquinolones and aminoglycosides with percent resistance of 26, 33 and 18 %, respectively. 56 % *S. pneumoniae* isolates were resistant against erythromycin, 27 % against chloramphenicol and 23 % against cefuroxime. The studies revealed that *S. aureus* and *S. pneumoniae* strains were high producer of biofilms which could be one of the reasons for their high pathogenicity.

## Introduction

Upper respiratory tract infections are one of the common diseases which can be found in the individuals of all age groups. Pharyngitis, nasopharyngitis, tonsillitis, otitis media and sinusitis are the major infections of upper respiratory tract. These major problems are generally caused by viruses (rhinovirus, adenovirus, parainfluenza virus, human metapneumovirus, and influenza virus. After the viral invasion, the secondary infection is caused by various types of bacteria resulting in chronic obstructive lung disease and high fever. The different types of bacteria which are involved in upper respiratory tract infections (RTIs) are *Haemophilus influenzae*, *Streptococcus pyogenes*, *Moraxella catarrhalis*, *Staphylococcus aureus*, and *Streptococcus pneumonia*. Although the upper respiratory tract infection cases are not complicated, the acute swelling caused by these infections may threaten airway patency resulting in asthmatic condition or cause obstruction in the passage of ingestion which further leads to significant dehydration. The RTIs are more common in developing countries in which, after diarrhea, RTIs are the second cause of death in children due to pneumonia (Ndip et al. 2009). Each year, United States reports that mortality rate of ∼14 % (5.6 million cases) is directly associated with community-acquired pneumonia (CAP) (Karchmer 2004).

Bacteria are becoming resistant to conventional antibiotics. Ten years ago, concern centered on Gram-positive bacteria, particularly methicillin-resistant *S. aureus* and vancomycin-resistant *Enterococcus* spp (Kumarasamy et al. 2010). Now, however, large number of clinical microbiologists agree that multidrug-resistant Gram-negative bacteria pose the greatest risk to public health (Ukpai et al. 2015). The increase in resistance of Gram-negative bacteria (faster than in Gram-positive bacteria) is higher than active new/developmental antibiotics, and only a few drug development programs are insufficient to provide therapeutic cover in next 10–20 years. The main reason for increase in resistance from antibiotics in bacteria is due to the mobile genes present on the plasmids which spread and transfer through bacterial populations (Bennett 2009). The overuse and misuse of antibiotics for the treatment of respiratory tract infection has been considered one of the major reasons for the emergence of resistance of bacteria against antibiotics (Gonzales et al. 1997). Another reason for antibiotic resistance of bacteria has been considered to be the production of biofilms during quorum-sensing-regulated mechanism which releases beta-lactamase responsible for degradation of various antibiotics (Wilke et al. 2005). It has been reported that *S. pneumoniae* were found to be resistant to penicillin and erythromycin, *H. influenzae* were resistant from ampicillin, whereas *S. pyogenes* isolates were resistant from erythromycin (Karchmer 2004).

Nowadays, the resistance of bacteria against existing antibiotics has been at alarming stage throughout the world with high mortality rate due to acute infections by various bacteria in respiratory infections in China, so more studies are required to isolate the novel bacterial strains resistant to various antibiotics. The pool of isolated resistant bacteria will be used to develop the potential strategy so as to target the regulation of bacterial resistance mechanisms to enhance the potency of available drugs, restore the efficacy of available drugs and develop the new range of antibiotics specific to new mechanism of drug resistance (Lister et al. 2009). In the present study, attempts were made to isolate the bacterial species from the infected persons of different age groups residing in different localities in China which were resistant against commonly prescribed antibiotics in China.

## Materials and methods

### Materials

Sterilized swabs were purchased from Fisher scientific (UK); Blood agar and eosin methylene blue agar were purchased from Sigma Aldrich (USA); for biochemical tests, API 20E kit was purchased from Biomerieux (France) and Mueller–Hinton plates were purchased from Biotech (England). All other laboratory reagents were purchased from Merck-Millipore Chemicals.

### Experimental designs and subjects

The studies were conducted in Guangdong Province, China. The province is thickly populated and is about 1902 m above the sea level. Average daily temperature in Guangzhou in January and July are 18 and 33 °C, respectively, with high humidity. The favorable temperature and relatively high humidity are mainly responsible for the respiratory tract infections.

The study was conducted on three different localities and three different age groups. The first group was having subjects with age ranging 5–25 years, which were mainly students studying in schools and colleges. The second group was 26–45 years age group which consists of working-class people and the third age group was ranging 46–65-year-old people. Totally, 900 subjects were selected (300 persons in each locality), and each locality includes 155 males and 145 female subjects. The subjects were informed prior about the aim of study, and their verbal consent was solicited. All the subjects were assured regarding privacy of disease and data. The three different localities were designated as L-1, L-2 and L-3. The different age groups, i.e., 5–25 years, 26–45 years and 46–65 year were designated as X, Y and Z, respectively. All the subjects included in the study were having symptoms of sore throat, cough, running nose, and the study was conducted between March 2014 and October 2014.

### Isolation of bacterial strains

Three hundred throat swabs from individuals of different age groups were taken in the sterilized plastic sample bags for the study. The samples were collected between 6 months (March 2014–August 2014) which included both dry and wet seasons which is favorable for the growth and invasion of various microorganisms.

The swabs were aseptically inoculated on the Petri plates containing blood agar media, and the plates were incubated at 37 °C in incubator for 24 h. After incubation, the morphological as well as microscopic examination of the colonies was done. The colonies obtained on the petri plates were subcultured on solid media several times to get the pure culture of the microorganisms. When the pure cultures were obtained, the microorganisms were subcultured on the solid media in slants and preserved at 4 °C for further investigations. Microorganisms in pure culture were identified on the basis of their morphological characteristics on selective and differential media. API 20E kits were used for biochemical testing to confirm the identity of microorganisms.

### Antibiotic susceptibility test

Antibiotic susceptibility test using microbroth dilution method was used to investigate the antibiotic susceptibility/resistance of isolates (Hendriksen 2003; Tilton et al. 1973). In brief, the test was carried out in a 96-well microtiter plate by distributing 240 µL of sterile media and 30 µL of antibiotic stock (5 mg mL^−1^) giving concentration of 500 µg mL^−1^; 150 µL from first well was transferred to the next and was diluted with sterile medial with factor of 2. Dilution up to 10 wells were giving the range of 500–0.197 µg mL^−1^, and appropriate blanks (to have a check on sterility) and controls (to see the growth-favoring ability of media) were also taken. To each well (except blank), 10 µL of isolated and identified bacterial inocula (10^7^ cells mL^−1^) were added, and plate was incubated in rotary shaker incubator 150 rpm, 30 °C for 36 h. The qualitative observation of turbidity was carried out with naked eyes under light and dark background and compared with clear blank well and hazy control well to make a decision of growth. The minimum inhibitory concentration (MIC) of particular antibiotic against particular organism was defined as lowest concentration where no viability of bacteria was observed in the wells of 96-microwell plates after incubation of 36 h.

### Detection of biofilm formation

Most of the antibiotic-resistant bacteria form biofilms during their growth and metabolism which are responsible for their resistance against many antibiotics. Two different biofilm formation analysis tests were performed. First test was based on the colorimetric detection of crystal violet-stained bacteria which is also known as tissue culture plate test (TCP) (Stepanovic et al. 2007). Another method for detection of biofilm was used which was based on the Congo red dye test (Freeman et al. 1989). In brief, the stock strain was transferred to Mueller–Hinton agar and was incubated for 24 h, 37 °C. Purified strain culture was resuspended in phosphate buffer pH 7.0 50 mM and diluted appropriately to get turbidity equivalent to 0.5 McFarland standard, i.e., 10^8^ CFU/mL. Sterile tryptic soy broth supplemented with 2.5 % glucose was taken for inoculation of isolated bacteria and were incubated for 24 ± 0.5 h at 37 °C with no shaking. Gentle washing was done with 300 µL phosphate buffer (pH 7.0, 50 mM) five times. Biofilm fixation was done through 150 µL methanol exposure for 15 min. The wall-fixed biofilm-containing bacteria was stained with 200 µL of 2 % crystal violet. After 15 min exposure, rest of the stain was removed and washed with buffer till no dye appeared in the wash. The well plate is dried at room temperature; then, the dried,stained,wall-fixed biofilm is solubilized by 95 % ethanol for 30 min, and OD was measured at 570.

## Results

### Bacterial isolates

The dominated species of pathogenic bacteria in three different localities of Guangdong Province of China has been presented in Table 1. From the various inoculated samples, 59 % of the samples examined were positive for infectious microorganisms. The four dominated species of pathogenic bacteria were identified as *H. influenzae*, *S. aureus*, *S. pneumoniae* and *Chlamydia pneumonia*. Each isolate/strain was given a code with respect to the species, e.g., H.I.1 for first isolate/strain of *H. influenza* and similarly for other species. Among four bacterial species, the highest number (23 %) of isolates of *H. influenzae* were obtained, whereas lowest number (8 %) were obtained of *C. pneumoniae*, whereas *S. aureus* and *S. pneumoniae* contributed 15 and 13 % of total isolates, respectively. It was observed that the maximum (64 %) number of isolates were obtained from locality-1 (L-1), whereas lowest isolates were found in the second locality (L-2).

**Table 1:** Different bacterial isolates in three different localities

Bacterial species	Locality	Number of isolates	Percentage (%) of isolates
*Haemophilus influenzae*	L-1	78	26
	L-2	45	15
	L-3	86	28.66
*Staphylococcus aureus*	L-1	41	13.66
	L-2	55	18.33
	L-3	38	12.66
*Streptococcus pneumoniae*	L-1	46	15.33
	L-2	32	10.66
	L-3	40	13.33
*Chlamydia pneumoniae*	L-1	28	9.33
	L-2	22	7.33
	L-3	20	6.66
Total		531	59

It was observed that among three different age groups, the maximum isolates (51.03 %) were obtained from the age group of 5–25 years among which 42 % of isolates were *H. influenzae* as shown in Table 2.

**Table 2:** Percentage of bacterial isolates found in three different age groups

Bacterial species	Age groups (years)							
	5–25		26–45		46–65		Total	
	Isolates (no.)	%	Isolates (no.)	%	Isolates (no.)	%	Isolates (no.)	%
*Haemophilus influenzae*	126	42	23	7.6	60	20	209	23.22
*Staphylococcus aureus*	63	21	30	10	41	13.66	134	14.88
*Streptococcus pneumoniae*	50	16.66	38	12.66	40	13.33	118	13.11
*Chlamydia pneumoniae*	32	10.66	18	6	20	6.6	70	7.77
Total	271	51.03	109	20.52	161	30.32	531	59

### Antibiotic susceptibility test

Microbroth dilution method was used to test the susceptibility of isolated microorganisms against different antibiotics such as ampicillin, amoxyclav, ciprofloxacin, chloramphenicol, erythromycin, cefuroxime and gentamicin ranging from (0.02–64 µg/mL), and criteria for suggesting whether the isolate is susceptible or resistant toward specific antibiotic have been given in Table 3. It was observed that the *H. influenza*e was highly resistant to ampicillin (43 % resistant strains) and erythromycin (41 % resistant strains), whereas it was least resistant to ciprofloxacin (9 % resistant strains) and gentamicin (12 % resistant strains) (Table 4).

**Table 3:** Interpretation of susceptible or resistant bacteria and the accepted range of MIC for the *E. coli* ATCC 25922 reference strain

Antibiotic	MIC interpretive criteria (µg/mL)		*E. coli* ATCC 25922. MIC (µg/mL)
	Susceptible (S)	Resistance (R)	
Ampicillin (AMP)	≤4	≥16	4
Amoxyclav (AMC)	≤1	≥4	1
Amoxicillin (AMO)	≤64	≥128	64
Ciprofloxacin (CIP)	≤0.08	≥0.32	0.08
Chloramphenicol (CHL)	≤4	≥16	4
Erythromycin (ERY)	≤0.2	≥0.8	0.2
Cefuroxime (CEF)	≤4	≥16	4
Gentamicin (GEN)	≤0.2	≥0.8	0.2

**Table 4:** Antibiotic resistance test of different bacterial isolates with different antibiotics

Isolates and number tested	No. of isolates resistant to antibiotics						
	Ampicillin	Amoxyclav	Ciprofloxacin	Chloramphenicol	Erythromycin	Cefuroxime	Gentamicin
*Haemophilus influenzae* (209)	90 (43 %)	48 (23 %)	25 (12 %)	56 (27 %)	85 (41 %)	32 (15 %)	18 (9 %)
*Staphylococcus aureus* (134)	87 (65 %)	62 (46 %)	35 (26 %)	44 (33 %)	72 (54 %)	52 (39 %)	24 (18 %)
*Streptococcus pneumoniae* (118)	63 (53 %)	45 (38 %)	50 (42 %)	32 (27 %)	66 (56 %)	27 (23 %)	25 (21 %)
*Chlamydia pneumoniae* (70)	40 (57 %)	16 (23 %)	12 (17 %)	38 (54 %)	44 (63 %)	12 (17 %)	14 (20 %)
Total	280 (53 %)	171 (32 %)	122 (23 %)	170 (32 %)	267 (50 %)	123 (23 %)	81 (15 %)

*Staphylococcus aureus* was resistant to β-lactam antibiotics such as ampicillin, amoxyclav as well as marcolide antibiotics such as erythromycin with percent resistance of 65, 46 and 54 %, respectively (Table 4). *S. aureus* was also moderately resistant to fluroquinolone antibiotics such as ciprofloxacin, chloramphenicol and aminoglycosides antibiotics such as gentamicin with percent resistance of 26, 33 and 18 %, respectively.

In this study, *S. pneumoniae* was observed to be 56 % resistant against erythromycin, 27 % against chloramphenicol and 23 % against cefuroxime (Table 4). It was observed that comparative to other isolates, *S. pneumoniae* was even 42 and 21 % resistant against ciprofloxacin and gentamicin which were found to be highly sensitive antibiotics against other isolates. *C. pneumoniae* was observed to be highly resistant against chloramphenicol and erythromycin compared to other antibiotics; it was having 57 and 63 % resistance against chloramphenicol and erythromycin, respectively (Table 4).

### Biofilm production test

**Fig. 1:**
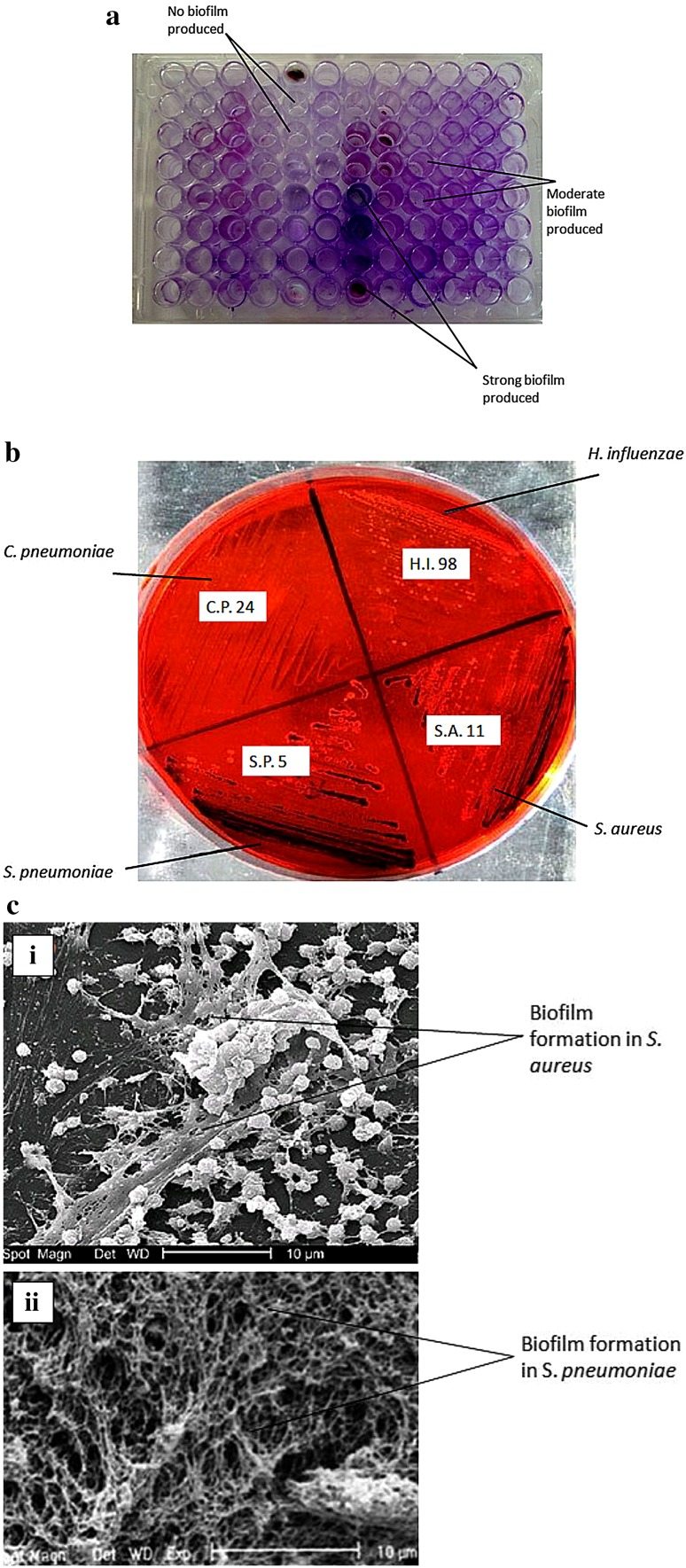
Tissue culture plate (TCP) test for biofilm production in bacterial isolates (**a**), Congo red dye test for biofilm production in isolates (**b**) and SEM images of biofilm produced in (*i*) *S. aureus* and (*ii*) *S. pneumoniae* (**c**)

Many pathogenic microorganisms form a slimy layer during invasion and infections which is basically termed as biofilm. All isolates were studied for biofilm production using tissue culture plate (TCP) test and Congo red dye test. Purple-bluish color was observed in the tissue culture plate wells when stained with gram stain. The organisms which produced dark purple-bluish color in tissue culture wells were considered as strong biofilm-producing strains, the bacteria with light purple-bluish color stained in wells were considered as moderate biofilm-producing strains, whereas the bacteria which were not stained in the wells were considered as no biofilm-producing strains (Fig. 1a).

It was observed that among four major isolates, *S. aureus* and *S. pneumoniae* were found positive for biofilm production. *S. pneumoniae* and *S. aureus* strains were observed to produce strong biofilms, whereas few strains of *H. influenza*e and *C. pneumoniae* were producing moderate biofilms with light purple color staining in the micro-titre plate wells and maximum strains were found to be negative for biofilm production with no purple color staining in the tissue culture plate wells. The strong and moderate biofilm-producing strains were further tested by Congo red dye test method in Petri plates. It was observed that the strong biofilm-producing S.A.11 strain of *S. aureus* and S.P.5 strain of *S. pneumoniae* were observed to produce dark black-colored colonies in congo red plate indicating strong biofilms production, whereas C.P.24 strain of *C. pneumoniae* and H.I.98 strain of *H. influenza*e which have shown moderate biofilm production in TCP test were able to produce black-colored colonies in congo red dye plate indicating no biofilm production (Fig. 1b). The SEM images of *S. aureus* and *S. pneumoniae* in Fig. 1c clearly showed the biofilm production in these strains.

## Discussion

From the results in Table 1, it was observed that the maximum (64 %) number of isolates was obtained from locality-1 (L-1), whereas lowest isolates were found in second locality (L-2). The possible reason for maximum isolates from L-1 could be the unhygienic living standards as well as damp and humid environment which were the favorable conditions for the growth and development of various pathogens. Moreover, the locality was thickly populated which had also increased the risk of communicable infections.

Table 2 shows bacterial isolates found in three different age groups, and depicts that *H. influenzae* was the most dominant during secondary respiratory tract infections in children. This age group included mostly the school/college going children. Many students were found to be underweight, malnourished and debilitated due to which they were having lower immunity and more vulnerable to the respiratory infections. The minimum isolates (20.52 %) were obtained from the age group of 26–45 years among which the 12.66 % isolates were *S. pneumoniae* as this age group was basically composed of the healthy adults working in hygienic conditions and found to have good immunity against infections. In the age group of 46–65 years which was mostly composed of old-age persons, 30.32 % of isolates were obtained with 20 % of *H. influenzae*.

Antibiotic susceptibility studies showed *H. influenza*e to be highly resistant to ampicillin and erythromycin, however, it was least resistant to ciprofloxacin and gentamicin. It has been reported that in *H. influenza*e, the resistance from ampicillin and amoxicillin was mediated by TEM-1 β-lactamase enzyme (Rennie and Ibrahim 2005). In previous studies, chloramphenicol was considered as the best antibiotic against *H*. *influenza*e, but few studies in north India revealed no resistance from this drug (Puri et al. 1999). Ampicillin inhibits the cell wall synthesis of bacteria by binding to penicillin-binding proteins (PBPs) so, maybe due to mutations or environmental changes in bacteria, ampicillin was not able to bind with the PBPs resulting in bacterial resistance to ampicillin (Sharma et al. 2013). Also, erythromycin was not able to inhibit the growth of *H. influenza*e even at higher concentrations which might be due to some protective mechanism that it developed against erythromycin. Few studies had also reported *H. influenza*e resistance toward ciprofloxacin due to mutations in the quinolone resistance-determining regions (QRDRs) of *gyrA* and *parC* (Brenwald et al. 2003). Ciprofloxacin inhibits the bacterial replication, transcription and repair by inactivating topoisomerase II (DNA gyrase) and topoisomerase IV enzymes. But in this study, *H. influenza*e showed no resistance against ciprofloxacin. Along with ciprofloxacin, gentamicin was also found to be effective against *H. influenza*e strains due to its irreversible binding to 30S units of bacterial ribosomes which inhibits its protein synthesis. Ciprofloxacin and gentamicin both were reported to be susceptible for *H. influenza*e in previous studies (Zanchi et al. 1994).

On the other hand, *S. aureus* was resistant to β-lactam antibiotics as well as marcolide antibiotics. Although *S. aureus* has broad-spectrum resistance against different class of antibiotics such as β-lactams, glycopeptides, quinolones, aminoglycosides, oxazolidinones, quinupristin–dalfopristin due to presence of various resistance genes (Lowy 2003), in another study, *S. aureus* was found to be resistant against methicillin antibiotics due to presence of *mec* and *ccr* genes (Chambers and Deleo 2010); but in this study, ciprofloxacin, chloramphenicol and gentamicin were found to be highly susceptible for *S. aureus* with low resistance. Due to the horizontal gene transfer of complex genetic array of *mec* element or vanA operon, it became resistant to different types of antibiotics (Pantosti et al. 2007). Cefuroxime was having moderate effect on *S. aureus* with 61 % sensitivity.

*S. pneumoniae* is a major causative agent in respiratory tract infections in humans, and they were once highly penicillin-susceptible microorganisms, but recently, they were found to be multidrug-resistant (Tomasz 1997). *S. pneumoniae* has been found to be responsible for estimated one million deaths each year in children below 5 years (Obaro et al. 1996). Susceptibility of *S. pneumoniae* was studied in Canada, and it was observed that ciprofloxacin was having poor potency against *S. pneumoniae* (Patel et al. 2011). After 10 years of use in United States, the susceptibility of ciprofloxacin was examined with *S. pneumoniae*, and the MIC was observed to be increased due to bacterial resistance (Sahm et al. 2000).

*C. pneumoniae* was observed to be highly resistant against chloramphenicol and erythromycin in this study. In previous studies, it was observed that *C. pneumoniae* is susceptible to erythromycin, azithromycin, roxithromycin, clarithromycin, doxycycline, ofloxacin, and rifampin, and their combination is more lethal (Freidank et al. 1999) which was in agreement with our study.

## Conclusion

The study revealed that various pathogenic bacteria in respiratory tract infection had become resistant to wide range of commonly used antibiotics. *H. influenzae* was the major causative agent for respiratory infections found in all age groups as its maximum number of isolates was obtained in the present study. Although considerable percentage of all isolates were observed to be resistant to various antibiotics, *S. aureus* and *S. pneumoniae* were found to be highly resistant to strong antibiotics like ciprofloxacin and gentamicin which might be due to the presence of resistant genetic elements present in these strains. The emergence of highly resistant bacteria against the commonly prescribed antibiotics is at alarming level; therefore, novel antibiotics should be introduced for severe respiratory tract infections. Moreover, different antibiotics in combination can be helpful against various resistant pathogenic strains.

In few studies, *H. influenza*e has been reported to produce strong biofilm (Galli et al. 2007; Mizrahi et al. 2014), but in the present study, strong biofilm production in *H. influenza*e strains was not observed. One of the possible reasons for biofilm production in various bacteria is quorum sensing. Quorum sensing in *S. aureus* was found to be responsible for the production of biofilms during various infections, and gene regulator *agr* was found to play important role in its biofilm production (Yarwood et al. 2004). The production of biofilms in bacteria has been reported to enhance the bacterial resistance against antibiotic, for example, production of biofilms in *S. aureus* was responsible for its resistance against methicillin (McCarthy et al. 2015). In another study, 60.47 % of *S. aureus* were found to be biofilm producers when tested with crystal violet test method, while 13.95 % of strains were found to be biofilm producer when tested with congo red agar (CRA) test method (Elkhatib et al. 2014) which showed high variability in these two methods which is in agreement with our study. In previous studies, *S. pneumoniae* was also observed to be good producer of biofilm during respiratory tract infections (Moscoso et al. 2006) which is in agreement with this study.
